# Efficacy of transcranial magnetic stimulation in borderline personality disorder

**DOI:** 10.1192/j.eurpsy.2025.1910

**Published:** 2025-08-26

**Authors:** M. Chelidze, M. Chelidze, M. Chelidze

**Affiliations:** 1 Psychiatry, Todua clinic, Tbilisi, Georgia

## Abstract

**Introduction:**

Transcranial Magnetic Stimulation is non-invasive neuromodulation technique that manages various mental disorders, including Borderline Personality Disorder (BPD) that is characterized by impulsive behavior, emotional lability, aggressiveness and unstable mood. TMS may decreases some core symptoms of borderline personality disorder by targeting specific brain regions involved in emotion regulation, aggression management and impulse control.

magnetic fields stimulate nerve cells in prefrontal cortex, involved in emotional regulation, also influences neural circuitry, that diminish symptoms like aggressiveness, dysregulation and problems in interpersonal relationships.

**Objectives:**

- **Treatment effectiveness assessment in reducing symptoms like problems in interpersonal relationships, mood swings, impulsivity, aggressiveness, sometimes paranoid ideations**
- **management of affect, anxiety and depressive states**
- **Changes in neural circuitry:** alterations in brain activity patterns
- **Safety profile** of TMS in individuals with BPD, also adverse reactions
- self assessment of the pationt regarding symptom relief
- **Compare with medications**

**Methods:**

**Type of Study:** Randomized controlled trial (RCT)

**Duration:**

10-14 weeks

**Setting:**

Outpatient department of Todua clinic

**Inclusion Criteria:**

- Adults aged 18-65 y
- Diagnosis of Borderline Personality

**Exclusion Criteria:**

- Contraindications (e.g., metal implants in the skull or brain)
- substance abuse disorder or psychiatric illness ( cognitive disorder, schizophrenia, bipolar disorder)

**Results:**

The results have shown a really promising outcome:

1. **reduced symptom severity: impulsivity, aggressiveness, self-harming, emotional instabiliyu, mood swings, improved mood and decreased anxiety and depressive states.**
2. **Changes in neuronal circuits:** TMS changed total brain activity in a positive way, it regulated limbic system functioning tha correspondingly improved the symptoms.
3. **This technique is really safe and very well tolerated with** few side effects, most of them were mild, such as headache.
4. **Improved Quality of Life**

**Conclusions:**

In conclusion, Transcranial Magnetic Stimulation leads to significant reductions in main symptoms, such as mood swings, impulsivity, aggressiveness, emotional dysregulation and self harm. moreover, this technique is very well-tolerated, with minimal side effects, like mild headache.

Changes in brain activity shows how TMS facilitates symptom improvement, especially when combined with psychotherapy or medications.

TMS promises to be an innovative technique nin the management of core symptoms of BPD and improving patients’ quality of life.

**Disclosure of Interest:**

None Declared

